# *Ureaplasma parvum* Serovar 3 Multiple Banded Antigen Size Variation after Chronic Intra-Amniotic Infection/Colonization

**DOI:** 10.1371/journal.pone.0062746

**Published:** 2013-04-26

**Authors:** James W. Robinson, Samantha J. Dando, Ilias Nitsos, John Newnham, Graeme R. Polglase, Suhas G. Kallapur, J. Jane Pillow, Boris W. Kramer, Alan H. Jobe, Diane Payton, Christine L. Knox

**Affiliations:** 1 Institute of Health & Biomedical Innovation, Faculty of Health, Queensland University of Technology, Brisbane, Queensland, Australia; 2 School of Women’s and Infants’ Health, The University of Western Australia, Perth, Western Australia, Australia; 3 Department of Neonatology and Pulmonary Biology, Cincinnati Children’s Hospital Medical Center, University of Cincinnati, Cincinnati, Ohio, United States of America; 4 School of Anatomy, Physiology and Human Biology, University of Western Australia, Perth, Western Australia, Australia; 5 Department of Pediatrics, School of Oncology and Developmental Biology, Maastricht University Medical Center, Maastricht, The Netherlands; 6 Pathology Queensland, Royal Brisbane and Women’s Hospital, Herston, Queensland, Australia; Université de Montréal, Canada

## Abstract

*Ureaplasma* species are the microorganisms most frequently associated with adverse pregnancy outcomes. The multiple banded antigen (MBA), a surface-exposed lipoprotein, is a key virulence factor of ureaplasmas. The MBA demonstrates size variation, which we have shown previously to be correlated with the severity of chorioamnion inflammation. We aimed to investigate *U. parvum* serovar 3 pathogenesis *in vivo*, using a sheep model, by investigating: MBA variation after long term (chronic) and short term (acute) durations of *in utero* ureaplasma infections, and the severity of chorioamnionitis and inflammation in other fetal tissues. Inocula of 2×10^7^ colony-forming-units (CFU) of *U. parvum* serovar 3 (Up) or media controls (C) were injected intra-amniotically into pregnant ewes at one of three time points: day 55 (69d Up, n = 8; C69, n = 4); day 117 (7d Up, n = 8; C7, n = 2); and day 121 (3d Up, n = 8; C3, n = 2) of gestation (term = 145–150d). At day 124, preterm fetuses were delivered surgically. Samples of chorioamnion, fetal lung, and umbilical cord were: (i) snap frozen for subsequent ureaplasma culture, and (ii) fixed, embedded, sectioned and stained by haematoxylin and eosin stain for histological analysis. Selected fetal lung clinical ureaplasma isolates were cloned and filtered to obtain cultures from a single CFU. Passage 1 and clone 2 ureaplasma cultures were tested by western blot to demonstrate MBA variation. In acute durations of ureaplasma infection no MBA variants (3d Up) or very few MBA variants (7d Up) were present when compared to the original inoculum. However, numerous MBA size variants were generated *in vivo* (alike within contiguous tissues, amniotic fluid and fetal lung, but different variants were present within chorioamnion), during chronic, 69d exposure to ureaplasma infection. For the first time we have shown that the degree of ureaplasma MBA variation *in vivo* increased with the duration of gestation.

## Introduction

The *Ureaplasma* species, bacteria from the class Mollicutes, are the most prevalent, potentially pathogenic bacteria isolated from the urogenital tract of both men and women (40–80%) [1] and are the microorganisms most frequently associated with preterm birth [2], [3], [4]. The ureaplasmas are among the smallest and simplest self-replicating prokaryotes, they do not possess a cell wall and are surrounded only by a plasma membrane [5]. Ureaplasmas have a small genome, between 750 kb and 1.2 M/bp in size. They have reduced biosynthetic capabilities and are therefore dependent on an animal host for survival [6], [7], [8].

The two species of ureaplasmas known to colonize humans are *U. urealyticum* (serovars 2, 4, 5, 7–13) and *U. parvum* (serovars 1, 3, 6 and 14). Of these, *U. parvum* is the most common species isolated from the genital tracts of men and women [1], [9], [10]. Ureaplasma colonization of the female upper genital tract in pregnancy is associated with preterm labor, preterm birth, perinatal morbidity and mortality, premature rupture of membranes [8], [11], [12] and is a major cause of histological chorioamnionitis [1], [13], [14]. Ureaplasma infections are often clinically asymptomatic and the incidence is higher in women who deliver preterm [3]. Intrauterine inflammation and ureaplasma colonization induce fetal lung maturation prematurely, predisposing the infant to the future likelihood of chronic lung disease (CLD) [15]. Isolation of ureaplasmas from endotracheal secretions of newborns [16] shows that infection of the fetus can occur *in utero* or alternatively be acquired by vertical transmission at birth [17].

Ureaplasma infection in animal models has been associated previously with dramatic variation in the histological inflammation of infected tissues [18]. Variable inflammation may be due to different or inconsistent interactions between the ureaplasmas and the host [15], [18], [19]. Previously, *Ureaplasma* spp. were investigated using a sheep model of long term (chronic) *in utero U. parvum* infection. This study demonstrated that variation of the surface-exposed ureaplasma multiple banded antigen (MBA) of serovar 6 correlated with the extent of chorioamnion inflammation [18]. The severity of chorioamnionitis correlated inversely with the number of MBA/multiple banded antigen gene (*mba*) size variants that existed within infected amniotic fluid (AF), suggesting that variation of the MBA/*mba* was associated with ureaplasmal pathogenicity [18], [20]. However, the relationship between the number of MBA antigenic variants and the severity of inflammation within infected chorioamnion and other infected tissues has not been investigated for *U. parvum* serovar 3, or during an acute duration of infection.

The 5′ region of the *mba* encodes a conserved N-terminal anchor of the lipoprotein whereas the 3′ region of the *mba* encodes the C-terminal domain, consisting of multiple tandem repeat units, which are surface-exposed. The C-terminal domain is antigenic and elicits an antibody host response during ureaplasma infection [21], [22]. Additions or deletions in the number of repeat units in the downstream region of the *mba* is associated with antigenic variation [21].

Our research group has utilised an ovine model to investigate the response of the fetus to a number of *in utero* challenges, including intra-uterine infection. The perinatal sheep model offers several advantages: the size of the fetus and fetal tissues are very similar to that of human fetuses and therefore the consequences of *in vivo* infection can be compared when investigating the effects on tissues. Furthermore, sheep do not deliver before 125d after intra-amniotic inoculation with ureaplasmas [23], thus providing an important control when investigating infection of different durational periods. For this study, *U. parvum* serovar 3, the most common serovar isolated from both males and females [1], was injected into pregnant ewes at one of three time points throughout the gestational period. We hypothesised that exposure of fetal tissues to either chronic or acute durations of ureaplasma infection would result in marked differences in MBA size variation between treatment groups and that this would correlate with varied inflammatory and histological responses within the fetal tissues.

## Materials and Methods

Out bred pregnant sheep (n = 32) were assigned to six experimental groups for this project. This study was carried out in accordance with the National Health and Medical Research Council ‘Australian code of practice for the care and use of animals for scientific purposes’ and approved by The University of Western Australia Animal Ethics Committee (Approval No. RA3/100/619).

*U. parvum* serovar 3, strain 442S (isolated originally from semen of infertile men attending the Wesley IVF Service [9]) was injected into the amniotic sac of ewes bearing singleton pregnancies. Using ultrasound guidance, AF was aspirated prior to inoculation and tested to confirm it was AF (and not allantoic fluid). Ureaplasma inocula of 2×10^7^ CFU were injected intra-amniotically at: day 55 of gestation (n = 8, term = 145–150 days gestation); day 117 (n = 8); and day 121 (n = 8). All fetuses were delivered surgically at 124 days (preterm). Controls groups included sheep inoculated with 10B media [24] at day 55 of gestation (n = 4); day 117 (n = 2) and day 121 (n = 2) of gestation.

### Delivery and Tissue Sampling

Ewes were anaesthetised, the fetuses were delivered surgically and samples of AF, chorioamnion, fetal lung (FL), umbilical cord (CORD) and cerebrospinal fluid (CSF) were collected aseptically for subsequent culture [18], [25]. The pH of AF and FL was measured. The deflation limb of a pressure-volume curve was performed to measure lung compliance up to a pressure of 40 cm H_2_O [2], [23]. Lung samples of the right upper lobe and right middle lobe were also collected aseptically for subsequent histological analysis and culture [2]. Specimens were either snap frozen in liquid nitrogen and stored (−80°C) or fixed in 4% formalin [2], [23].

### Culturing and Quantification

To detect ureaplasmas within AF, chorioamnion, CORD and FL, samples were cultured. Thawed chorioamnion, CORD and FL (0.1 grams) were homogenized and cultured as previously described [2], [18] in 10B broth and on A8 agar [26] to determine the number of CFU of ureaplasmas per gram of tissue or per mL of fluid. Broths then were incubated at 37°C, aerobically for 24–48 hours. *Ureaplasma* spp. growth was detected by an alkaline shift in the broth media due to the production of ammonia [26]. Agar plates then were incubated at 37°C, under 5% CO2 for 48–72 hours, and ureaplasma colonies were counted using a stereomicroscope (Leica Microsystems, North Ryde, NSW).

### Histology

The formalin-fixed chorioamnion, CORD and FL samples were paraffin embedded, and 5–10 µm sections cut. The sections were heated overnight at 60°C and then stained with haematoxylin and eosin (H & E). The stained tissues were examined blindly and the number of white blood cells (WBC): monocytes, neutrophils; and lymphocytes present in 20 microscopic fields per slide at ×1000 magnification were counted. The H & E tissue sections were visually examined by a perinatal pathologist, and graded according to the diagnostic criteria outlined by Redline *et al.* [27].

### Cloning and Filtration

Ureaplasmas isolated from selected FL samples from animals chronically colonized intra-amniotically with ureaplasmas were cloned and filtered twice, as previously described [18], [28], to obtain ureaplasma cultures originating from a single CFU. The FL tissues were selected from fetuses associated with chorioamnionitis of a mild grade, a severe grade and a third in which scar formation occurred within the chorioamnion. The original ureaplasma inoculum (442S) was also cloned and filtered as a control.

### DNA Extraction

Ureaplasma DNA was extracted and purified from AF and tissue homogenates, of chorioamnion, CORD and FL collected from all animals using the QIAamp DNA Mini Kit (QIAGEN Ltd, Crawley, UK) according to the manufacturer’s tissue protocol.

DNA was also obtained from each passage 1 (P1) culture for all tissues (AF, chorioamnion, CORD and FL) and from clone 2 (C2) FL ureaplasma cultures using previously described methods [29]. Briefly, 500 µL of cultures were centrifuged, the supernatants discarded, and the pellets each resuspended in 125 µL Solution A (10 mM Tris HCl pH 8.5, 100 mM KCl, 2.5 mM MgCl2), 125 µL Solution B (10 mM Tris HCl pH 8.5, 2.5 mM MgCl2, 1% v/v Tween 20, 1% v/v Triton X) and proteinase K (120 µg/mL). Specimens then were incubated at 60°C for 1 hour, then 94°C for 10 minutes, and then stored at −20°C.

### Polymerase Chain Reactions (PCR)

PCR primers were designed using the *U. parvum* serovar 3 reference serovar genome sequences; strain ATCC700970 [8], and the more recent ATCC27815 strain. The PCR primers, UMS3UF and UMS3UR (F^5′^TTACCAAATCTTAGTGTTC^3′^, R^5′^CTGGTTGTGTAGTTTCAAAG^3′^) amplified the conserved upstream region of the *mba*. Cycling involved an initial denaturing period at 95°C for 15 minutes, followed by 35 cycles involving: denaturation at 95°C for 1 minute, primer annealing at 54°C for 1 minute, extension at 72°C for 1 minute, and a final extension step at 72°C for 10 minutes. The downstream repeat region of the *mba* was amplified with designed primers UMS3DF (F^5′^CTTTGAAACTACACAACCAG^3′^) and UMS3DR (R^5′^TTCAGGTTTAAAAAATGGGC^3′^). For this reaction, cycling conditions involved an initial denaturing period at 95°C for 10 minutes, followed by 40 cycles of 95°C for 45 seconds, 52–54°C for 75 seconds, and 72°C for 2 minutes and a final extension step at 72°C for 15 minutes.

The PCR assay was performed in a total volume of 50 µL with final concentrations of 0.1 mM of dNTPs (Invitrogen, Mt Waverley, VIC), 1× PCR buffer (Tris HCL, KCL, (NH_4_)_2_SO_4_, pH 8.7; Invitrogen), 45 mM MgCl_2_ (Invitrogen), 0.5 µM of each primer (Sigma-Proligo, Castle Hill, NSW), 1U Platinum *Taq* (Invitrogen), 8 µL of template DNA, and 32 µL of UltraPure DNase/RNase-Free distilled water (Invitrogen). Positive controls for all PCR assays included the initial *U. parvum* serovar 3 inoculum (442S) and the *U. parvum* serovar 3 reference serovar corresponding to strain ATCC27815 (Courtesy of H. Watson, University of Alabama, Birmingham). Master-mix only and dH_2_O negative controls were also included in each PCR assay. PCR amplicons were analysed by electrophoresis using a 2% agarose tris borate EDTA (TBE) gel at 100 volts for 60 minutes and visualised by ethidium bromide staining.

### SDS PAGE and Western Blot

Ureaplasma P1 cultures from AF, chorioamnion, CORD and FL tissue, and C2 FL cultures were analysed by western blot analysis as previously described [18]. For this protocol the membranes were incubated with primary antibody diluted 1/10000 (polyclonal rabbit serum raised against *U. parvum* serovar 3, courtesy of Emeritus Dr Patricia Quinn, Toronto, Ontario). The positive controls for the western blots were cultures of the *U. parvum* serovar 3 initial inoculum (442S) and *U. parvum* serovar 3 reference serovar [8], [30]. A 10B media negative control was included to demonstrate any cross-reactivity of the antiserum with components of the 10B broth media.

### Statistical Analysis

Data are presented as mean ± standard error of the mean (SEM). One-way analysis of variance (ANOVA) was used to analyse the numbers of ureaplasma in AF (CFU/mL) and chorioamnion, CORD, and FL tissues (CFU/g) and to examine differences between chronic and acute ureaplasma treatment groups. Two-way ANOVA was used to analyse differences between treatment cohorts, inflammatory cell counts and inflammatory cell type. Univariate random effect ANOVA using the general linear model was used to analyse FL pressure-volume curves (PV curves) for differences between treatment groups, ureaplasma infection duration and pressure, *p* values of ≤0.05 were considered statistically significant.

## Results

### Fetus Delivery

Pregnancy losses included two fetuses from ewes injected intra-amniotically with ureaplasmas at 55 days gestation and two fetuses from ewes that were injected at 117 days gestation. There was also one pregnancy loss from a control animal injected with media at 121 days of gestation. A final count of 27 singleton fetuses were sampled across the 6 experimental groups.

### Culturing and Quantification

Ureaplasmas were cultured from 100% of AF, 90% of FL, 65% of chorioamnion and 35% of CORD tissue samples from animals that were injected intra-amniotically with ureaplasmas. No ureaplasmas were detected in the tissues from animals injected with the media control. A total of 80 passage 1 (P1) ureaplasma cultures were obtained from these tissues for further analysis. Ureaplasmas were not detected in any CSF specimens by culture or PCR assay. The mean ureaplasma titre (CFU/mL) was higher (>10^6^ CFU per mL) in the amniotic fluid of animals across all treatment groups (*p*<0.007) when compared to ureaplasma titres in the other tissues collected from the same animal. Differences were not statistically significant for the other tissues and the different treatment groups.

### pH Analysis of Amniotic Fluid (AF) and Fetal Lung (FL) Fluid

**Figure 1:**
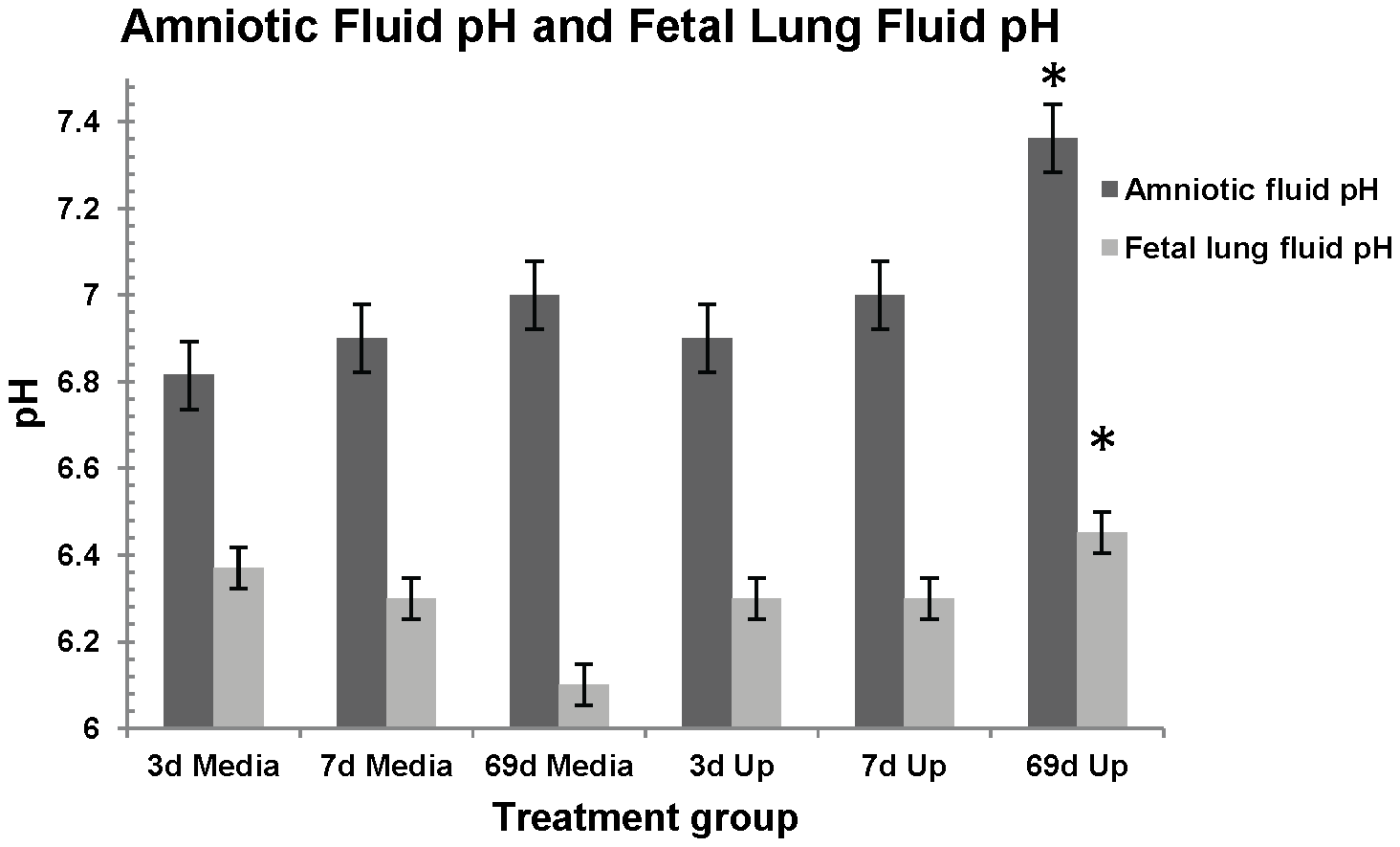
Amniotic fluid and fetal lung fluid pH analysis. AF from 69 day chronic ureaplasma exposed animals showed a higher pH when compared to control animals (*p = 0.03) and 3 day acute animals (*p = 0.02). FL fluid from 69 day chronic ureaplasma exposed animals also showed a statistically higher pH (*p = 0.0058) when compared to the pH of FL from7d and 3d acute animals and control animals.

The mean AF pH was higher in animals exposed to ureaplasma infection from 55 - 124 days (69 days) of gestation (pH = 7.36±0.1) compared to the pH of AF from control animals (pH = 6.9±0.1) (*p* = 0.03) and AF of animals exposed to acute ureaplasma infection (3 days, pH = 6.9±0.1, *p* = 0.02). No AF pH differences were observed between any of the acute or control treatment groups (Figure 1).

The FL fluid pH was also higher in animals exposed to chronic ureaplasma infection (pH = 6.5±0.1) than for animals exposed to ureaplasmas for 7 days (pH = 6.3±0.1), 3 days (pH = 6.3±0.1) or control animals (pH = 6.3±0.1) (*p* = 0.0058). There were no significant FL fluid pH differences between animals exposed to ureaplasmas for 7 days or 3 days and control animals.

### Lung Pressure-Volume Curve

**Figure 2:**
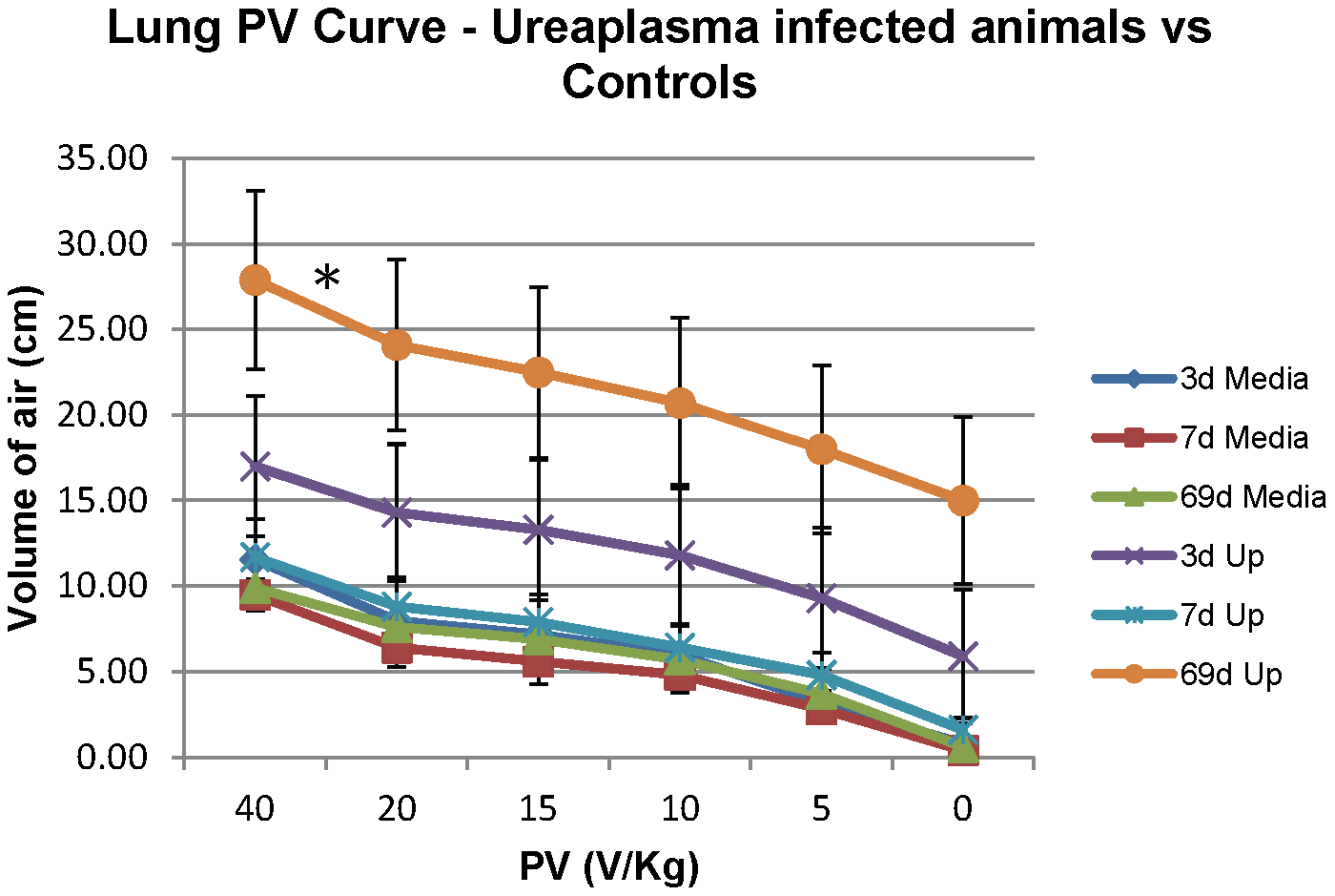
Lung Pressure-Volume (PV) curve, performed on fetal lungs at time of preterm delivery. Animals exposed to chronic (69d) ureaplasma infection (* *p* = 0.0365) showed greater lung compliance in comparison to the media control group; the 3d acute (*p* = 0.165) and the 7d acute (*p* = 0.49) treatment groups.

Animals exposed to chronic (69d) ureaplasma infection (*p* = 0.0365) showed greater mean lung compliance in comparison to the media control group; and also when compared to the 3d acute (*p* = 0.165) and the 7d acute treatment groups (*p* = 0.49) (Figure 2).

### Histology

The number of inflammatory cells (neutrophils, monocytes, and lymphocytes) in the chorioamnion tissues from animals injected with ureaplasmas was compared to the cell numbers in animals inoculated with media control. Surprisingly, there were no differences in inflammatory cell numbers within the chorioamnion of animals injected with ureaplasmas and the chorioamnion tissue from the media control groups (*p* = 0.278). Whilst neutrophils counts were higher than the other inflammatory cell types observed in the tissue (i.e. monocytes and lymphocytes), this difference was not significant (*p* = 0.068).

**Figure 3:**
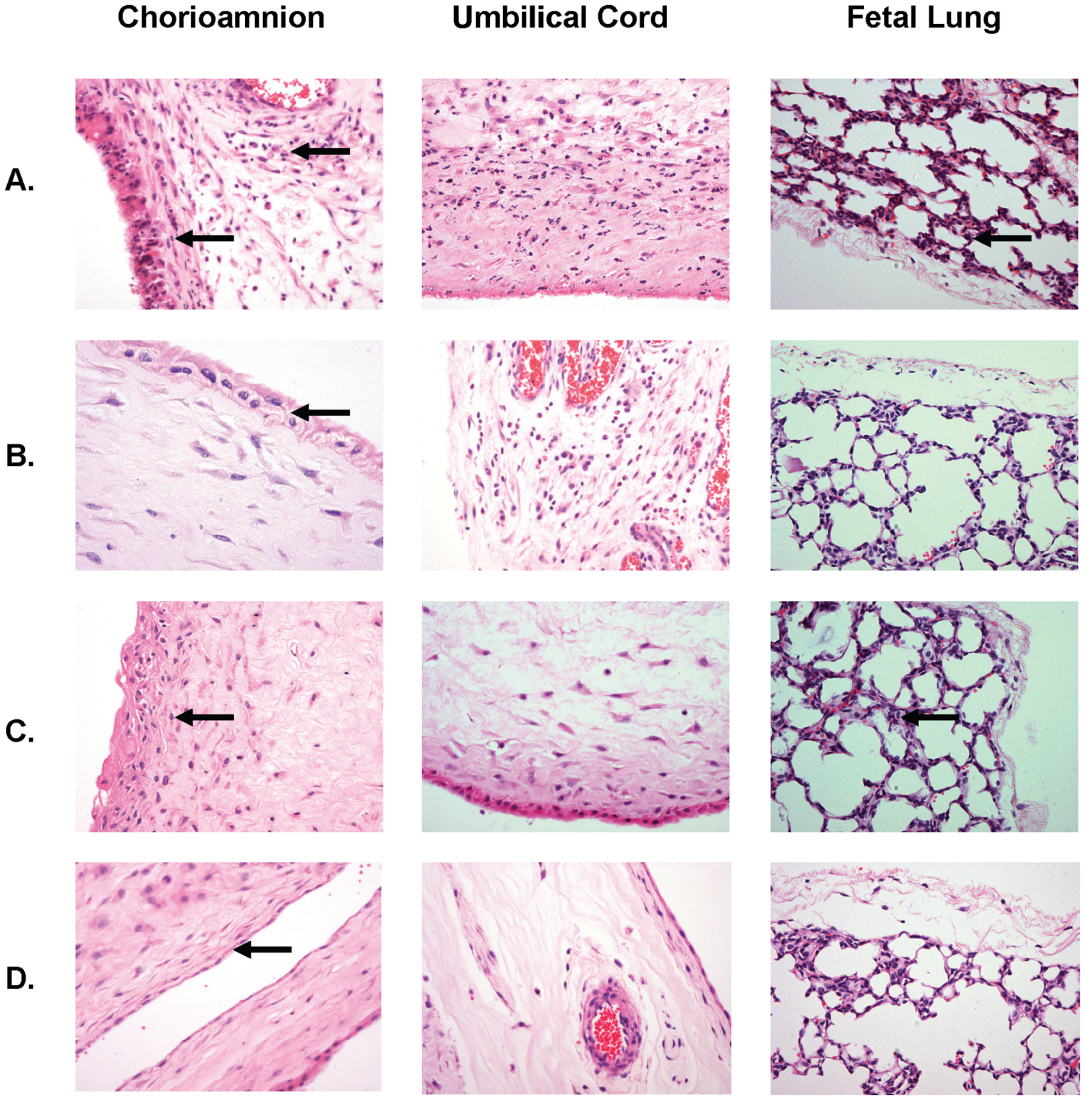
H & E staining of tissues selected from three animals exposed to chronic, 69d, intra-amniotic ureaplasma infection. Variable levels of inflammation were observed in chorioamnion, umbilical cord, and FL tissues. These animal tissue images were selected based on the severity of inflammation within the chorioamnion: A: severe chorioamnionitis; B: mild chorioamnionitis; C: scarring of chorioamnion; and D: Uninfected (control), minimal inflammation. The severity of inflammation observed in the FL and CORD tissues corresponded consistently with the severity of histological chorioamnionitis.

The amniotic fluid and fetal lung pH values were also correlated with the inflammatory cell counts within the chorioamnion and fetal lung H & E tissue sections. However, elevated pH within these body fluids was not associated with elevated inflammatory cell counts (R^2^<0.04). Variability in the severity of inflammation was observed in tissues of animals exposed chronically to ureaplasmas. The appearance of chorioamnion tissues exposed to chronic (69d) ureaplasma infection ranged from severe inflammation with thickened epithelial membranes, irregular shaped epithelial cells and infiltration of WBCs throughout the collective tissues layers (Figure 3A- arrowed), to mild inflammation with thickening of epithelium cells (Figure 3B); or to chorioamnion with fibrous scar tissue and degraded architecture (Figure 3C). In the chorioamnion, from control animals no thickening of the epithelium and only mild inflammatory changes were observed (Figure 3D). The FL tissue appearance also ranged from mild inflammation (Figure 3B), similar in appearance to the control tissue (Figure 3D); to severe inflammation with an influx of WBCs into the tissue (Figure 3A); to a high degree of fibrosis and scarring (Figure 3C). CORD tissue exposed to chronic (69d) ureaplasma infection also demonstrated mild inflammation with low numbers of WBCs present (Figure 3B); to severe inflammation with high WBC counts in the tissues (Figure 3A). CORD tissue also demonstrated fibrosis and scarring (Figure 3C). The variable degrees of inflammation observed in the FL and CORD tissues correspond consistently with the severity of histological chorioamnionitis observed in the chorioamnion tissues from the same animals.

### Summary of Pathology

Pathology results using the Redline classifications [27] summarised in Table 1, demonstrated that the severity of inflammation of the maternal and fetal chorioamnion membranes increased with the duration of ureaplasma colonization. Interestingly acute chorioamnionitis, a stage 2 maternal response (Table 1) was present in all of the control animal tissues as well as the tissues infected with ureaplasmas for 3d, 7d, or 69d. This maternal response may indicate an inflammatory response to components within the 10B media. However, the increased duration of exposure to intra-amniotic ureaplasmas was associated with an influx of macrophages and neutrophils within the maternal membranes of the chorioamnion when compared to the response within the control tissues (Maternal response Stage 2/Grade 1(b) – Table 1).

**Table 1:** Maternal/fetal chorioamnion tissue inflammatory responses for pregnant ewes colonized intra-amniotically with ureaplasmas for 3 days, 7 days or 69 days compared to the responses within control animal chorioamnion tissues.

SeverityMaternal^1^ response			Duration of intra-amniotic ureaplasma colonization		
Stage	Grade	Control	3 Days	7 Days	69 Days
		n animals 3 (%)	n animals (%)	n animals (%)	n animals (%)
**2**	**1(a)**	6 (86%)	5 (62.5%)	3 (50%)	1 (16.5%)
**2**	**1(b)**	1 (14%)^4^	3 (37.5%)	3 (50%)	4 (67%)
**3**	**2**				1 (16.5%)
**Severity** **Fetal response** 2					
**Stage**	**Grade**	**Control**	**3 Days**	**7 Days**	**69 Days**
**0**	**0**	6 (86%)	6 (75%)	2 (33.5%)	1 (16.5%)
**1**	**1**	1 (14%)^4^	1 (12.5%)	3 (50%)	4 (67%)
**1**	**2**		1 (12.5%)	1 (16.5%)	1 (16.5%)
**Total animals**		**7**	**8**	**6**	**6**

1 maternal inflammatory responses as stage 1 (early; acute subchorionitis or chorionitis), stage 2 (intermediate; acute chorioamnionitis), and stage 3 (advanced; necrotizing chorioamnionitis), Grade 1: mild-moderate; (a) with 1+ macrophages within the chorion; (b) with 2+ macrophages and ≥1+ neutrophils within the chorion; Grade 2: severe.

2 fetal inflammatory responses as stage 1 (early; chorionic vasculitis or umbilical phlebitis), stage 2 (intermediate; umbilical vasculitis, one or two arteries, and/or vein or umbilical panvasculitis, all vessels), and stage 3 (advanced; [subacute] necrotizing funisitis or concentric umbilical perivasculitis). Grade 0: nil; 1: mild-moderate; Grade 2: severe. (Redline *et al.* 2003).

3 Control animals injected with media intra-amniotically: 3d (n = 1), 7d (n = 2) and 69d (n = 4) prior to surgical delivery.

4 a 69d control animal.

By contrast, only one (14%) of the control tissues (a 69d control) demonstrated a fetal membrane inflammatory response within the chorioamnion compared to 25% (3d), 66.5% (7d) and 83.5% (69d) of the tissues exposed to ureaplasmas (Fetal response Table 1).

### Analysis of Passage 1 (P1) Cultures - *Western Blot Analysis*

**Figure 4:**
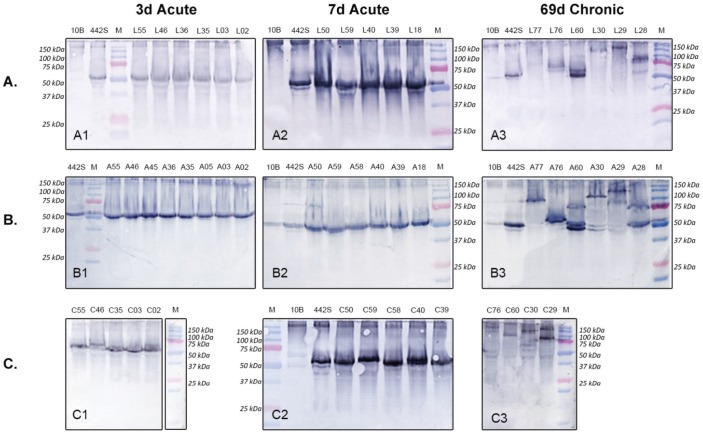
P1 Western blot demonstrating MBA antigenic variation. Western blots comparing antigenic variation of P1 ureaplasma isolates from animals colonized/infected with *U. parvum* serovar 3 for 69 days (chronic infection), 7 days and 3 days (acute infection). The number of antigenic variants (single bands) within A. FL (L samples), B. AF (A samples), and C. chorioamnion (C samples) P1 cultures are compared. 442S = serovar 3 initial inoculum control; 10B = 10B media negative control; M = Precision Plus Dual Colour Protein Standard (BioRad, Gladesville, NSW).

Western blot analysis of P1 ureaplasma cultures obtained from FL, AF and chorioamnion tissues (of animals exposed to acute 3d intra-amniotic ureaplasma infection) demonstrated no size variation of the MBA in comparison to the 442S original *U. parvum* serovar 3 inoculum (50 kDa) (Figure 4 A1, B1, C1). After 7 days *in vivo* P1 ureaplasmas demonstrated minor size variation of the MBA in isolates from the FL/chorioamnion (Figure 4 A2, C2). By contrast, dramatic size variation of the MBA antigen was observed in P1 ureaplasmas from the FL, AF and chorioamnion of animals chronically infected/colonized with ureaplasmas (from day 55 of gestation) (Figure 4 A3, B3, C3).

A comparison of the MBA variation within AF and FL tissues collected from the same animals (which were inoculated at 55 days of gestation) demonstrated common MBA variants within these tissues: sample A76 and L76 (Figure 4 A3, B3) both showed two comparable MBA size variants (60 kDa and 55 kDa); sample A60 and L60 (Figure 4 A3, B3) both showed three comparable size variants (75 kDa, 60 kDa and 50 kDa); samples A28 and L28 (Figure 4 A3, B3) both showed two comparable size variants (75 kDa and 50 kDa); and samples A30 and L30 (Figure 4 A3, B3) both showed one comparable size variant (100 kDa) (NB - lower bands in A30 are due to overflow from A60). However, there were exceptions: for L77 there was no band but a band was present in A77 (Figure 4 A3, B3); and A29 demonstrated two size variants (150 kDa, 90 kDa) but L29 showed only one variant (150 kDa) (Figure 4 A3, B3). However, the pattern of variation observed in the AF and FL tissues from the same animal was not consistent with the variation observed in the corresponding chorioamnion (Figure 4 C3). Unfortunately, no ureaplasmas were cultured from CORD specimens that were exposed to chronic ureaplasma infection.

PCR assays were performed on each of the P1 and clone 2 clinical ureaplasma isolates. PCR assays of the downstream region of the *mba* gene demonstrated size variation of the *mba* (results not shown) and the *mba* size variants detected by PCR correlated directly with the western blot MBA size variants.

### Fetal Lung Clone 2 Analysis - *Western Blot Analysis (Fetal Lung C2)*

**Figure 5:**
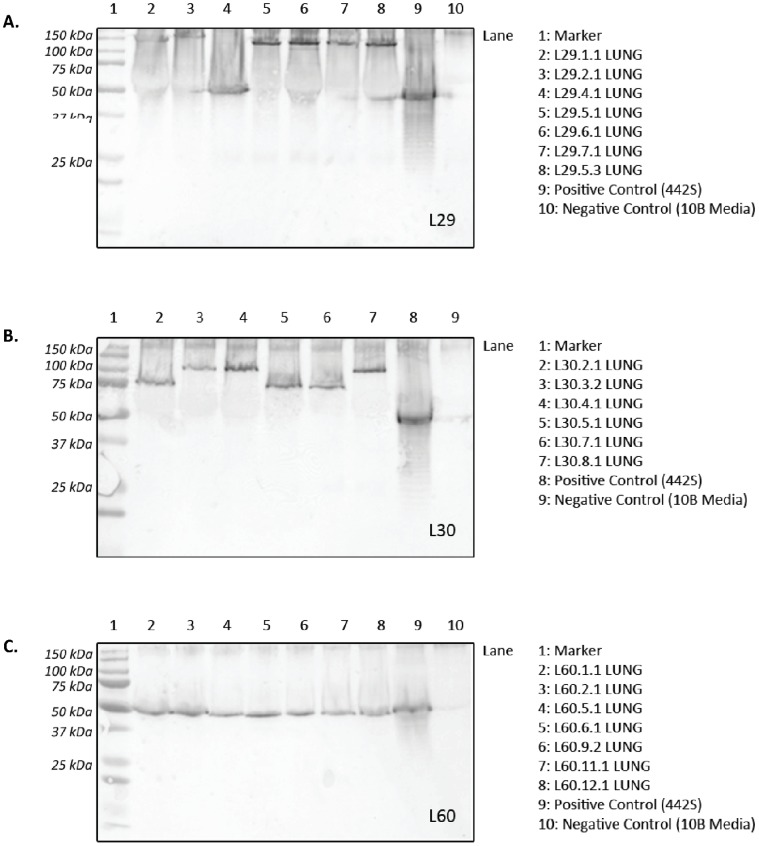
Western blots of FL clone 2 ureaplasma isolates. A: In specimen L29, 3 ureaplasma size variants (150 kDa, 140 kDa, 50 KDa) were detected in this severely inflamed lung tissue; B: Specimen L30: Mildly inflamed tissue demonstrating 2 size variants (105 kDa and 75 kDa); C: L60: Scarred tissue demonstrating only 1 size variant (50 kDa). 442S = serovar 3 initial inoculum control; Marker = Precision Plus Dual Colour Protein Standard (BioRad, Gladesville, NSW). The numbers identify the lung tissue (L29, L30 and L60) and the C2 isolate number.

Cloning and filtering was performed on selected FL tissues (L60, L30 and L29), those chronically exposed to ureaplasmas for 69 days *in vivo*, to obtain cultures from a single CFU. Seven C2 isolates for each FL tissue then were tested by western blot, and *mba* PCR (data not shown). Each C2 ureaplasma isolate demonstrated only a single MBA variant (Figure 5A, B and C).

The C2 isolates from the severely inflamed FL tissue, L29 (Figure 5A), demonstrated 3 size variants (150 kDa, 140 kDa, 50 kDa) and these variants also demonstrated the greatest size variation of the MBA when compared to the initial inoculum (442S). The C2 isolates from the mildly inflamed FL tissue, L30 (Figure 5B), demonstrated 2 size variants (105 kDa and 75 kDa). By contrast, clones isolated from scarred FL tissue, L60 (Figure 5C), demonstrated only one MBA variant (50 kDa) by western blot analysis and this was equivalent in size to the 442S (ureaplasma inoculum) variant.

## Discussion

This study investigated the effects of high dose (2×10^7^ CFU) *U. parvum* serovar 3 on the preterm sheep fetus after intra-amniotic infection/colonization and different durations of infection. Importantly we demonstrated dramatic MBA size variants within the AF, FL and chorioamnion, that were directly associated with the duration of *in vivo* ureaplasma colonization. Ureaplasmas cultured from animals inoculated with ureaplasmas at day 121 (3d acute) and day 117 (7d acute) showed little or no variation of the MBA. By contrast, the MBA of ureaplasmas isolated from animals inoculated with ureaplasmas at day 55 of gestation (69d chronic) showed a large degree of MBA size variation. Zheng *et al.* [20], [21] demonstrated MBA variation among four clinical isolates (each from a single CFU) obtained from an ureaplasma culture from an infected FL specimen of a newborn infant: the clinical ureaplasmas cultured contained a mixture of *mba* size variants. More recently, we showed that variation of the surface exposed antigen (MBA) of ureaplasma occurred *in vivo* in an animal model after chronic durations of infection and that variation of the antigen correlated directly with variation of the *mba* gene [18]. In this current study we show for the first time that the degree of ureaplasma MBA variation *in vivo* increased over the duration of infection/gestation. Both this study and our previous study [18]; show that fewer MBA variants are generated *in vivo* by serovar 3 in contrast to serovar 6 [18]. Furthermore, whilst serovar 6 generated both larger and smaller size variants in comparison to the original inoculum used in the study (*U. parvum* serovar 6, 306S) [18], only larger MBA variants were generated by serovar 3 (*U. parvum* serovar 3, 442S, this study and [18]). Size variation of the MBA may be a mechanism that ureaplasmas utilise to evade immune pressures from the host (the innate and acquired immune responses) [18], [21], [25]. However, we showed recently, that size variation of the MBA did not directly contribute to the severity of inflammation and chorioamnionitis [25]. Instead, we proposed that variation of the surface exposed MBA antigen may prevent the eradication of ureaplasmas by the host immune response.

After short term (acute 3d and 7d) durations of ureaplasma infection *in vivo*, there was insufficient time to generate the numerous MBA size variants that were observed in isolates obtained from animals exposed to chronic ureaplasma infection. Bacterial surface exposed antigen(s) often contain pathogen-associated molecular patterns (PAMPs) that host cells and receptors, such as Toll-like receptors (TLR), recognise [31]. Shimizu *et al*. [32] demonstrated that the MBA is a major virulence factor of *U. parvum* and is recognised by TLR1, TLR2 and TLR6, inducing an inflammatory response. The development of a specific antibody response is an important component of the host defence against many mycoplasmal diseases, including ureaplasmas [22], [33]. The host immune system requires time to generate a specific immunity to foreign antigens. Previously, we demonstrated an influx in inflammatory cells (neutrophils and monocytes) within fetal bronchoalveolar lavage fluid after 3, 6 or 10 weeks of *U. parvum* serovar 3 intra-amniotic colonization, but not after 1 week [23]. This inflammatory cell influx is consistent with reports of the first antibodies in sheep being produced after 7–14 days of exposure to infection [34], [35]. In this current study, we observed little or/no variation in the MBA during the early exposure of 3d/7d acute ureaplasma infection. By contrast, in the chronic 69d ureaplasma infection group, the host immune response and immunoreactivity would be well established [23], [25] and in this animal cohort dramatic variation in the MBA was observed. These observations further support the hypothesis that interactions between ureaplasma organisms and the host immune response stimulate variation within the MBA [15], [19], [23], [25]. Our study, together with these earlier findings, suggests that size variation of the MBA may be a mechanism by which ureaplasmas alter the PAMPs contained in this surface antigen and this may enable the ureaplasmas to evade the host immune system.

The pattern of MBA variation observed in the current study was not always conserved between different tissues from the same animal. Ureaplasma isolates obtained from chorioamnion specimens showed variation of the MBA, which was different to the MBA variation observed in isolates from AF and FL tissues, in which identical variants were detected in the same animal. These results are not surprising as the AF and FL are contiguous *in utero* and therefore ureaplasmas were exposed to the same immune pressures from the host. The divergent variation of the ureaplasma MBA observed in the chorioamnion tissue demonstrates that ureaplasmas were exposed to different selective pressure in this different tissue compartment [36]. Microbes have the ability to adapt to anatomical differences (e.g. chorioamnion versus FL/AF), regulating expression patterns/virulence factors in response to different environmental cues (e.g. osmolarity, pH, oxygen, or ions) [37]. Furthermore, these expression patterns evolve/change over time [38], suggesting another reason for the differences observed in ureaplasma MBA variation.

We also observed an increase in the severity of inflammation within the chorioamnion associated with an increase in the duration of exposure to intra-amniotic ureaplasmas (Table 1). However, there was also variability in the severity of inflammation within chorioamnion tissues from animals exposed to chronic ureaplasma infection (69d). Knox *et al*. showed that ureaplasmas have the ability to chronically colonize the AF without inducing histological chorioamnionitis in some animals [18]. In the current study, only minimal inflammation was observed in chorioamnion tissues collected from some animals, which were colonized with ureaplasmas *in utero* for 69 days; for example, the chorioamnion tissue (Figure 3B), showed little change/inflammation when compared to the control chorioamnion tissue (Figure 3D). By contrast, severe chorioamnionitis (Figure 3A) and inflammation with scarring (Figure 3C) was observed in other animals. Furthermore, there were large variations in the inflammatory cell counts within the chorioamnion specimens collected from animals in each treatment cohort. Higher WBC counts were detected in chorioamnion tissues chronically exposed to ureaplasmas but the variability in counts between animals in each treatment group produced large error bars and as a result there was no significant difference found. The variability in histological presentation and the inflammatory cell counts in the chorioamnion tissues chronically exposed to ureaplasmas provide further evidence that in some ureaplasma infections the host immune response is actively avoided.

Previously, Knox *et al*. [18] demonstrated that size variation of the MBA of *U. parvum* serovar 6 correlated directly with size variation of the downstream repeat region of the *mba* in the sheep model. In this study we compared MBA and downstream *mba* size variation of P1 and C2 ureaplasmas cultured from fetal tissues and confirmed by PCR and western blot that the size variability of the MBA, the expressed lipoprotein, correlated with the size variation of the downstream repeat region of the *mba* gene.

Our results demonstrated increased lung compliance and maturation of the fetal preterm lung as a result of chronic (69d) intra-amniotic *U. parvum* serovar 3 infection but not after infection of ≤1 week. Previously, preterm fetal lung maturation was also observed in this sheep model [18] in animals infected chronically after intra-amniotic inoculations with serovar 6 [18] or serovar 3 [18], [23]. This suggests that while the mechanism of MBA/*mba* variation may differ between ureaplasma serovars, the resulting pathological effect on the preterm fetal lung is similar. Preterm fetal lung maturation was also observed previously after intra-amniotic administration of *Escherichia coli* endotoxin [23], [39]. These experimentally induced intra-amniotic infections/inflammations resulted in increased pulmonary surfactant within preterm lungs and improved lung function (lung compliance) in preterm lungs, which is consistent with the reduced risk of respiratory distress syndrome observed for preterm infants exposed to intra-uterine infection prior to birth [40].

The differences observed between *U. parvum* serotypes and their corresponding pathogenicity *in vivo* may be attributed to the mechanism(s) ureaplasmas utilise to vary the *mba*. A number of mechanisms can be employed by microbes to generate gene size variation, such as site-specific DNA rearrangements and gene conversion mechanisms [41]. DNA size variation can occur from the expansion or contraction of the number of repeat units through the mechanism of slipped-strand mispairing (SSM). SSM involves the misalignment of the repeat sequences between the daughter and parent strands during chromosomal replication or DNA repair. Misalignment of these strands can occur on the leader or lagging strand at the repeat region, resulting in an increase or decrease in the number of repetitive units in the newly synthesised DNA [29], [41], [42], [43]. SSM-mediated variation demonstrates repeat instability and allows for different combinations of variants to be expressed simultaneously [41], [42] as we have observed in the *mba* downstream repeat region of ureaplasma isolates cultured from animals exposed to chronic (69d) infection. Therefore, we propose that SSM mediated variation is the predominant mechanism utilised by ureaplasmas to alter the number of repeating units of the downstream region of the *mba.*

Analysis of the pH of amniotic fluid and fetal lung fluid at delivery (124d) demonstrated that chronic exposure to ureaplasmas resulted in an alkaline shift. Ureaplasmas hydrolyse urea as their sole source of energy, resulting in ammonium ions as a by-product of hydrolysis. The accumulation of ammonia after 69d of ureaplasma colonization resulted in the pH shift in the AF and the FL fluid. An alkaline environment inhibits the growth potential of ureaplasmas *in vitro* [44], [45], [46]. The pH of the environment *in utero* is therefore likely to be a limiting factor of ureaplasma growth. Ammonia reacts with water in tissues to form the strong alkali, ammonium hydroxide, which at high concentrations can cause chemical burns and damage the respiratory epithelium [47], [48]. Chronic lung disease after exposure to ammonia has been reported in adults [49]. We showed that an elevated pH in either the amniotic fluid or fetal lung fluid did not correlate with increased inflammatory cell counts in the chorioamnion or fetal lung tissue. However, in the presence or absence of inflammation, the ammonia liberated by ureaplasmas may contribute to the chronic tissue damage and pathology observed within the chorioamnion and the fetal lung *in utero.* The significance of association between ureaplasmas and preterm birth highlights the need to investigate the pathogenicity of ureaplasma colonization *in utero* and the association with adverse pregnancy outcomes. Our study has demonstrated for the first time, that ureaplasma MBA variation occurs after 7 days of *in vivo* ureaplasma colonization and this supports the proposal that variation occurs in response to the host immune system particularly as different MBA variants were detected in AF and fetal lung compared to those generated within the chorioamnion. The mechanism of MBA variation may therefore be a means by which ureaplasmas evade the host immune response and may also account for the pathological differences observed in the tissues from animals in each treatment group. Alternatively, the variation of the surface exposed MBA antigen may prevent the eradication of ureaplasmas by the host immune response [25]. We have progressed understanding of the link between MBA variation in *U. parvum* serovar 3. Further investigations of potential mechanisms enabling ureaplasmas to elude host immune responses will contribute to the understanding of ureaplasma pathogenicity and their role in preterm birth.
